# Defects in COG-Mediated Golgi Trafficking Alter Endo-Lysosomal System in Human Cells

**DOI:** 10.3389/fcell.2019.00118

**Published:** 2019-07-03

**Authors:** Zinia D’Souza, Jessica Bailey Blackburn, Tetyana Kudlyk, Irina D. Pokrovskaya, Vladimir V. Lupashin

**Affiliations:** Department of Physiology, University of Arkansas for Medical Sciences, Little Rock, AR, United States

**Keywords:** COG complex, golgi apparatus, CRISPR, endosomes, glycosyltransferase, GARP complex, endocytosis

## Abstract

The conserved oligomeric complex (COG) is a multi-subunit vesicle tethering complex that functions in retrograde trafficking at the Golgi. We have previously demonstrated that the formation of enlarged endo-lysosomal structures (EELSs) is one of the major glycosylation-independent phenotypes of cells depleted for individual COG complex subunits. Here, we characterize the EELSs in HEK293T cells using microscopy and biochemical approaches. Our analysis revealed that the EELSs are highly acidic and that vATPase-dependent acidification is essential for the maintenance of this enlarged compartment. The EELSs are accessible to both *trans*-Golgi enzymes and endocytic cargo. Moreover, the EELSs specifically accumulate endolysosomal proteins Lamp2, CD63, Rab7, Rab9, Rab39, Vamp7, and STX8 on their surface. The EELSs are distinct from lysosomes and do not accumulate active Cathepsin B. Retention using selective hooks (RUSH) experiments revealed that biosynthetic cargo mCherry-Lamp1 reaches the EELSs much faster as compared to both receptor-mediated and soluble endocytic cargo, indicating TGN origin of the EELSs. In support to this hypothesis, EELSs are enriched with TGN specific lipid PI4P. Additionally, analysis of COG4/VPS54 double KO cells revealed that the activity of the GARP tethering complex is necessary for EELSs’ accumulation, indicating that protein mistargeting and the imbalance of Golgi-endosome membrane flow leads to the formation of EELSs in COG-deficient cells. The EELSs are likely to serve as a degradative storage hybrid organelle for mistargeted Golgi enzymes and underglycosylated glycoconjugates. To our knowledge this is the first report of the formation of an enlarged hybrid endosomal compartment in a response to malfunction of the intra-Golgi trafficking machinery.

## Introduction

Membrane trafficking is a conserved and tightly controlled process in all eukaryotic cells transporting about 30–50% of total proteins synthesized in a cell (Bonifacino and Glick, 2004). Trafficking events, modulated by components of the trafficking machinery which include coat proteins, adaptors, small GTPases, coiled-coil tethers, MTCs, SNAREs and SNARE-associated proteins, must act in concert to ensure proper synchronization of all the steps involved. MTCs achieve this through their many orchestrated interactions with the trafficking machinery (Oka and Krieger, 2005; Yu and Hughson, 2010). The major MTC at the Golgi is called the COG complex and is conserved from yeast to humans, as its name implies (Miller and Ungar, 2012; Climer et al., 2018a). This octameric complex made of two distinct subcomplexes with four subunits in each sub-complex (so called lobes A and B) and involved in Golgi retrograde trafficking (Lupashin and Ungar, 2008; Willett et al., 2013b; Ha et al., 2016). Mutations in 7 out of 8 individual subunits give rise to congenital disorders of glycosylation (CDG) called COG-CDG, a type-II CDG associated with high morbidity and mortality (Wu et al., 2004; Foulquier et al., 2006; Kranz et al., 2007; Paesold-Burda et al., 2009; Reynders et al., 2009; Lubbehusen et al., 2010; Kodera et al., 2015). In agreement with observed CDG human disease phenotype, siRNA-modulated depletion of COG subunits in human cells resulted in both N- and O-glycosylation defects (Zolov and Lupashin, 2005; Shestakova et al., 2006; Pokrovskaya et al., 2011). Similar glycosylation defects and depletion of multiple Golgi glycosyltransferases have been observed in HEK 293T cells completely depleted for each of the individual COG subunits using a CRISPR-Cas9 approach (Bailey Blackburn et al., 2016). It was also noted that defects in glycosylation are only one important facet of COG deficiency. The COG complex functionally interacts with multiple components of the cellular trafficking machinery, including SNAREs (Shestakova et al., 2007; Laufman et al., 2011; Kudlyk et al., 2013; Willett et al., 2013a; Willett et al., 2016), Rabs (Miller et al., 2013), coat proteins (Suvorova et al., 2002), coiled-coil tethers (Sohda et al., 2007; Sohda et al., 2010; Miller et al., 2013), the Biogenesis of Lysosome-Related Organelles Complex 1 (BLOC-1) (Gokhale et al., 2012) and copper transporter ATP7A (Comstra et al., 2017). Disruption of specific protein-protein interactions in COG-deficient cells is likely to produce multiple mutant phenotypes. Indeed, in addition to glycosylation defects, COG deficient cells exhibited a disrupted Golgi morphology, altered secretion, mislocalization of various protein and lipids and the formation of vacuole-like enlarged-endolysosomal structures (EELSs) (Bailey Blackburn et al., 2016; Blackburn et al., 2018). Importantly, all these defective phenotypes appeared to be independent from well-described glycosylation defects and therefore require special investigation (Blackburn et al., 2018).

In this report, we have used microscopic, biochemical and genetic approaches to characterize in detail one of the most prominent phenotypes in COG-depleted cells – EELSs.

## Materials and Methods

### Reagents and Antibodies

Reagents were as follows: LysoSensor Yellow/Blue DND-160 (Life Technologies; L7545), LysoTracker Red DND-99 (Life Technologies; L7528) GNL-Alexa 647 (Willett et al., 2013c), Baf A1 (RP1, B40500), Filipin (Sigma-Aldrich; F4767), TopFluor-Cholesterol (Avanti Polar Lipids). Primary antibodies used for western blotting (WB) or immunofluorescence microscopy (IF) were made in the lab or commercially purchased. Antibodies and their dilutions were as follows: rabbit monoclonal anti-MGAT1 (Abcam; ab180578; WB 1:500), goat affinity purified polyclonal anti-B4GALT1 (R&D Biosystems; AF3609; WB 1:500), goat affinity purified polyclonal anti-ST6GAL1 (R&D Biosystems; AF5924; WB 1:500), β-Actin (Sigma; WB 1:1000), rabbit anti-COG4 (Ungar et al., 2002), rabbit polyclonal anti-VPS54 (St. John’s Laboratory; STJ115181; WB 1:1000), mouse anti-Lamp2 (DHSB; IF 1:100). Secondary antibodies used for WB or IF were as follows: fluorescent dye conjugated AffiniPure Donkey anti-mouse, anti-rabbit, or anti-sheep (IF 1:1000, Jackson Laboratories) and infrared dye IRDye 680RD or IRDye 800CW anti-mouse, anti-rabbit or anti-goat (WB 1:20,000, LI-COR).

### Cell Culture

HEK293T cells (ATTC) were grown in DMEM/F12 medium (Thermo Fisher Scientific) supplemented with 10% FBS (Atlas Biologicals, Lot #F07J17A1; Cat #F-0500-A) with or without antibiotic/antimycotics where indicated. Cells were grown at 37°C and 5% CO_2_ in a 90% humidified incubator. For passaging cells, they were briefly trypsinized (0.25% trypsin EDTA, Gibco) for 3–4 min and resuspended in media.

HEK293T COG1 through COG8 knockout clones were described previously (Bailey Blackburn et al., 2016; Blackburn and Lupashin, 2016; Climer et al., 2018b).

Both VPS54 knockout (KO) and VPS54/COG4 double knockout (DKO) cell lines were generated using CRISPR (Jinek et al., 2012; Cong et al., 2013; Mali et al., 2013) in a similar fashion in HEK293T or HEK293T COG4 KO cells. First, HEK293T-Cas9 stable cell line was created by lentiviral transduction with FLAG tagged Cas9. HEK 293 FT cells were used to produce lentiviral particles with lentiCas9-Blast and helper plasmids according to the protocol described by the manufacturer. Prior to transfecting HEK 293 FT cells, they were placed in serum free Opti-MEM with 25 μM Chloroquine and GlutaMAX. The next day, the media was replaced with Opti-MEM supplemented with GlutaMAX. After 48 h of transfection, the media was collected and cell centrifuged at 400 g for 10 min. The cell free supernatant was then filtered using a 0.45 μm PES filter. 1 ml of this filtrate was added to HEK293T or HEK293T COG4 KO cells seeded on 6 cm dishes. 24 h later the media was replaced with DMEM/F12 supplemented with 10% FBS and 10 μg/ml blasticidin. To knockout VPS54 in HEK293T-Cas9 cells, CRISPR dual gRNAs were purchased from Transomics (TEDH-1088059, TEDH-1088060, TEDH-1088062). HEK293T-Cas9 cells were transfected with a cocktail of these three gRNAs effectively inducing six cuts in VPS54 gene at the following target sequences:

Guide# TEDH-1088059 target sequences:

grna-a: ACAAATATTCCTGAAACAGGCAGAAGGAAC

grna-b: ATCTAGAAAGTGTTATGAATTCCATGGAAT

Guide# TEDH-1088060 target sequences:

grna-a: CAAAAGATAATTCACTGGACACAGAGGTGG

grna-b: CATTCTACCTCCCACAGATCAGCAAGGAAC

Guide# TEDH-1088062 target sequences:

grna-a: CTTAACTCTGTAGCCACAGAAGAAAGGAAA

grna-b: GTAAGCATGTCAGTAGTAACAGATGGGATG

Lipofectamine 3000 (Thermo Fisher Scientific) was used for transfecting cells according to the manufacturer’s protocol. VPS54 KO cells were selected with puromycin (3 μg/mL) for 2 days post-transfection. Surviving cells were then single cell plated onto a 96-well plate. Clonal populations of VPS54 KO were screened by western blot for absence of the targeted protein.

VPS54/COG4 DKO cells were created in HEK293T COG4 KO-Cas9 cells by transfecting them with the same VPS54-specific dual gRNAs used to create the single VPS54 KOs. VPS54/COG4 DKO cells were selected with puromycin (3 μg/mL) for 2 days post-transfection followed by single-cell plating onto 96-well plates to obtain clonal populations that were screened for absence of VPS54 gene product by western blotting.

### Plasmid Preparation and Transfection

Mammalian expression constructs were generated using standard molecular biology techniques or obtained as generous gifts. See Table 1 for complete list of plasmids. Plasmids were isolated from bacteria using the QIAprep Spin Miniprep Kits (Qiagen). Plasmid transfections were performed using Lipofectamine 3000 (Thermo Fisher Scientific) according to the manufacturer’s instructions.

**Table 1:** Plasmids used in this study.

CD63-GFP	Paul Luzio	Addgene plasmid # 62964; http://n2t.net/addgene:62964; RRID:Addgene_62964
lentiCas9-Blast	Feng Zhang	Addgene plasmid # 52962; http://n2t.net/addgene:52962; RRID:Addgene_52962 Sanjana et al., 2014
GFP-Rab1	Mitsunori Fukuda	Fukuda et al., 2008
GFP-Rab39a	Mitsunori Fukuda	Fukuda et al., 2008
GFP-Rab4	Mitsunori Fukuda	Fukuda et al., 2008
GFP-Rab7a	Mitsunori Fukuda	Fukuda et al., 2008
GFP-Rab9a	Mitsunori Fukuda	Fukuda et al., 2008
YFP-GOSR1	Rainer Duden	Willett et al., 2013a
GFP-SNAP23	Marc Coppolino	Kean et al., 2009
YFP-SNAP29	Rainer Duden	Willett et al., 2013a
GFP-Stx8	This lab	Liu et al., 2016
GFP-Stx16	This lab	Willett et al., 2013a
GFP-Stx17	Ghanshyam Swarup	Muppirala et al., 2011
GFP-Sec22	Rainer Duden	Willett et al., 2013a
GFP-Vamp3	Thierry Galli	Galli et al., 1998
GFP-Vamp4	Thierry Galli	Mallard et al., 2002
GFP-Vamp7	Thierry Galli	Martinez-Arca et al., 2000
GFP-Vamp8	Thierry Galli	Paumet et al., 2000
Lamp2-GFP	Santiago Di Pietro	Ambrosio et al., 2012
Lamp2-mCh	Santiago Di Pietro	Ambrosio et al., 2012
ST-RFP	Grégory Lavieu	Lavieu et al., 2013
TGN38-GFP	George Banting	Reaves et al., 1993
GFP-2×P4M	Sergio Grinstein	Levin et al., 2017
GFP-PH-Gab2	Sergio Grinstein	Levin et al., 2017
mRFP-FYVE-EEA1	Sergio Grinstein	Levin et al., 2017
mRFP-PH-PLCδ	Sergio Grinstein	Levin et al., 2017
Str-KDEL_SBP-mCh-Lamp1	Juan Bonifacino	Chen et al., 2017

### Transfection and Live Cell Immunofluorescence Microscopy

Cells were plated on collagen (50 μg/ml) coated 35 mm dishes glass bottom dishes with no. 1.5 coverglass (MatTek corporation), Lipofectamine 3000 was used to transfect cells as per the protocol described by the manufacturer. Briefly, the DNA and lipid reagent were separately diluted in Opti-MEM. Prior to transfection they were combined and added to dishes. Cells were incubated overnight with the DNA-lipid complexes and imaged the next day. Prior to imaging, the media was replaced with warm FluoroBrite DMEM Media (Life Technologies) supplemented with 10% FBS. Cells were imaged on an LSM880 Zeiss inverted microscope outfitted with confocal optics with the 63× oil 1.4 numerical aperture (NA) objective and Airyscan. The environment throughout imaging was controlled at 37°C, 5% CO_2_, and 90% humidity.

### BSA and Transferrin Labeling and Uptake

BSA (Sigma, #A7906) and Tf (EMD Millipore, #616397) were labeled using LI-COR’s IRDye 650 Protein Labeling Kit and VRDye 549 Protein Labeling Kit, respectively, according to the manufacturer’s protocol. Briefly, BSA or Tf was dissolved in azide free phosphate buffer, pH 8.5 to obtain a final concentration of 1 mg/ml. The labeling dye was dissolved in ultrapure water and added to the protein solution and the labeling was carried out for 2 h at room temperature in the dark. Pierce Polyacrylamide Spin Desalting Columns included in the kit were used to separate unconjugated dye after the labeling reaction was complete. The dye to protein ratio was calculated using the formula,

$$\frac{D}{P}=\frac{\mathrm{Absmax}}{\varepsilon \mathrm{Dye}}/\frac{A280-(\mathrm{CF}*\mathrm{Absmax})}{\varepsilon \mathrm{Protien}}$$

and was found to be 0.96 and 0.77 for BSA and Tf, respectively. HEK 293T COG4 KO cells were plated on 35 mm collagen coated glass bottom dishes. Cells were transfected with Lamp2-GFP to label the EELSs. The next day, they were incubated with 100 μg/ml BSA-650 and Tf-550 diluted in 10% FBS supplemented media for 30 min or overnight. After incubation, the cells were washed with DPBS six times, the media was replaced with 10% FBS and GlutaMAX supplemented FluoroBrite DMEM Media and the cells were imaged on the LSM880 as described above.

### Immunofluorescence Microscopy

12 mm glass coverslips (#1, 0.17 mm thickness) were collagen coated. HEK293T WT and COG4 KO cells were plated to be 60–70% confluent at the time of processing. Cells were washed with Dulbecco’s phosphate buffered saline (DPBS) and stained according to the protocol described previously (Zolov and Lupashin, 2005). Briefly, freshly prepared 4% paraformaldehyde (PFA) (16% stock solution diluted in DPBS; Electron Microscopy Sciences) was used as a fixative. After fixation, cells were permeabilized with 0.1% Triton X-100 for 1 min followed by treatment with 50 mM ammonium chloride for 5 min to quench free aldehydes. Cells were then washed three times with DPBS and blocked twice for 10 min each with 1% BSA, 0.1% saponin in DPBS. Antibodies were diluted in DPBS with 1% cold fish gelatin and 0.1% saponin. Cells were incubated with the primary antibody for 1 h at room temperature. Cells were washed four times with DPBS and incubated for 30 min with fluorescently tagged secondary antibodies diluted in antibody buffer. Cells were washed four times with DPBS. Hoechst diluted 1:10000 in DPBS was used to stain the nucleus. Coverslips were then washed four times with DPBS, rinsed with ddH2O, and mounted on glass microscope slides using Prolong Gold anti-fade reagent (Life Technologies). Cells were imaged with the 63× oil 1.4 numerical aperture (NA) objective of the LSM880 described above.

### High Pressure Freezing, Freeze Substitution, and Electron Microscopy

Electron microscopy was performed as previously described (Bailey Blackburn et al., 2016). Briefly, cells were plated on sapphire disk to be at 100% confluency next day. Disks were placed in PBS with 2% agarose, 100 mM D-mannitol, and 2% FBS then subjected to high pressure freezing using a Leica EM PACT2 with rapid transfer system. Samples were placed in acetone with 2% Osmium tetroxide, 0.1% Glutaraldehyde, and 1% ddH_2_O in liquid nitrogen then transferred to a freeze substitution chamber at −90°C. Cells were slowly warmed to 0°C and stained with a 1% Tannic acid/1% ddH_2_O solution in acetone for 1 h followed by staining with a 1% osmium tetroxide/1% ddH_2_O solution in acetone. Samples were then embedded in Araldite 502/Embed 812 resin (EMS) with DMP-30 activator added and processed in a Biowave at 70°C under vacuum for 3 min per embedding step. Samples were then baked at 60°C for 48 h before holders were removed and samples were cut. Post-cutting staining was done with uranyl acetate and lead citrate prior to imaging.

Ultrathin sections were imaged at 80 kV on a FEI Technai G2 TF20 transmission electron microscope. Images were taken with a FEI Eagle 4 k× USB Digital Camera.

### Retention Using Selective Hooks (RUSH) Assay

The Lamp1 RUSH reporter was a kind gift from Dr. Juan Bonifacino. The RUSH assay was performed as previously described (Boncompain et al., 2012; Chen et al., 2017). Briefly, cells were grown on collagen coated 35 mm glass dishes to a confluency of 60–70%. Prior to transfection, the media was supplemented with 100 μg/ml avidin to prevent biotin in the media from interfering with the RUSH reporter. Cells were co-transfected with the Lamp1 RUSH reporter and Lamp2-GFP using Lipofectamine 3000 as described above. After overnight transfection, the media was replaced with the chase mix which consisted of biotin and cycloheximide diluted to a final concentration of 40 μM and 50 μM, respectively, in FluoroBrite DMEM Media supplemented with 10% FBS and GlutaMAX.

To block the exit from the TGN, cells were incubated at 20°C for 90 min immediately after the addition of the chase mix. After 90 min incubation at 20°C, GNL-Alexa 647 (1:500) (Willett et al., 2013c) was added to the media and incubation was continued for another 30 min at 20°C effectively resulting in a total of 2 h incubation at 20°C which led to the retention of the RUSH reporter in the *trans*-Golgi upon its exit from the ER. The cells were washed 4–5 times with DPBS then, warm FluoroBrite DMEM Media supplemented with 10% FBS and GlutaMAX was added and the cells were imaged at 37°C.

### Drug Treatment, Cell Lysis and Western Blotting

Cells were grown to a confluency of 80% on 6-well plates and treated with 200 nM Baf A1 for 24 h. Following drug treatment, the cells were stained with Hoechst for 20 min and imaged on a Zeiss Axiovert 200 microscope with phase-contrast. For western blot analysis, cells were untreated or treated with 200 nm Baf A1 for 24 h. Before lysing the cells, media was removed, and the wells were washed three times with DPBS. Hot 2% SDS was added to the wells and the lysates were collected in microcentrifuge tubes which were immediately placed in a heating block at 70°C for 5 min. Amount of protein was determined using the Pierce BCA Protein Assay Kit as per the protocol. 6× sample buffer containing 5% β-mercaptoethanol was added to the lysates and 10 μg protein was loaded onto BioRad 4–15% gradient gels and the gel was run at 160 V.

An Invitrogen Power Blotter was used to transfer proteins onto a nitrocellulose membrane by semi-dry transfer. After transfer was complete, the membrane was washed with PBS and incubated for 30 min with Odyssey Blocking Buffer (LiCor). Primary antibodies were diluted in the same blocking buffer and were added to the membrane. The membrane was incubated overnight with primary antibody. The next day the membrane was washed three times and incubated with Donkey anti-Mouse IRDye 680RD or Donkey anti-Rabbit or Donkey anti-Goat IRDye 800CW secondary antibodies diluted in 5% evaporated milk in 1× PBS. The membrane was washed four times with 0.1% Tween-20 in PBS. The membrane was then air-dried and imaged using the Odyssey CLx Imaging System (LiCor). Analysis was done in Image Studio Version 5.2.

### Data and Statistical Analysis

The EELSs in phase contrast images were counted using the Particle Analysis plugin in ImageJ after setting an appropriate threshold to mask the background. Bar graphs representing the number of EELSs per cells were plotted in Microsoft excel 2010. The diameter of Lamp2 positive compartments in HEK293T WT and COG4 KO cells was manually estimated using Zen blue lite 2.3. Colocalization analysis was performed using the RGB color plugin in ImageJ which measured the signal intensities of mCh and GFP along a line of interest. For statistical analysis, Student’s *t*-test. was performed using the online “QuickCalcs” calculator of GraphPad Prism. Graphs were generated in Microsoft excel 2010.

## Results

### COG Depletion Results in the Accumulation of EELSs

**FIGURE 1:**
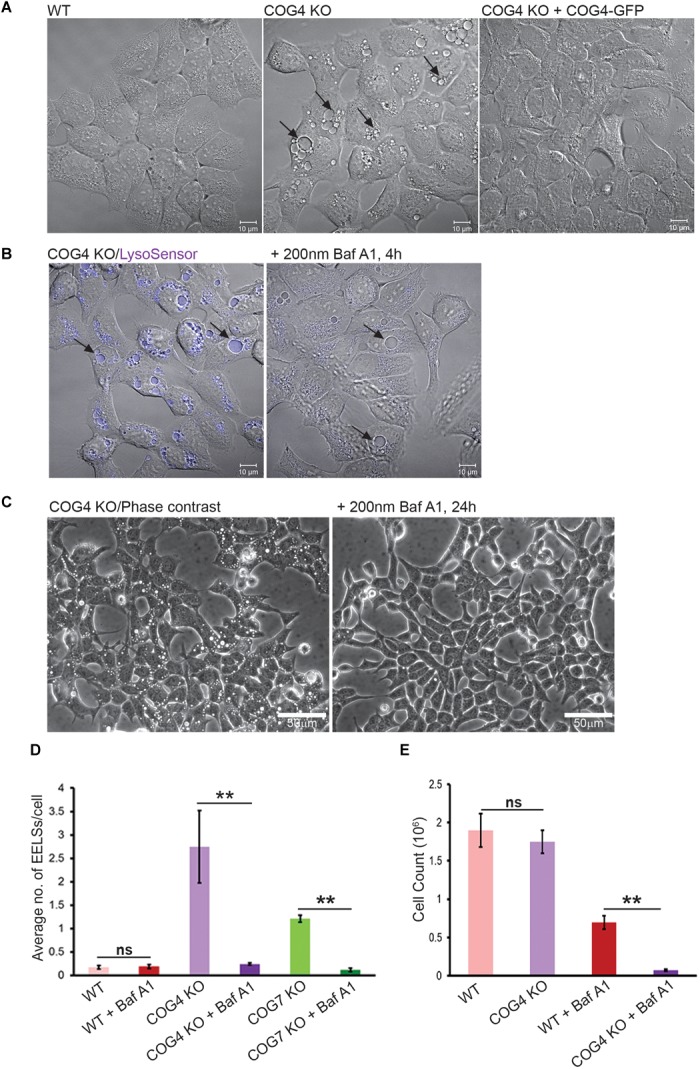
EELSs in COG KO cells are highly acidic and the activity of vacuolar ATPase is necessary for their long-term stability. **(A)** Specific accumulation of EELSs in COG KO cells. DIC images of HEK293T WT, COG4 KO, and stably rescued COG4 KO cells. **(B)** EELSs are highly acidic. HEK293T COG KO cells were incubated with LysoSensor Yellow Blue DND-160 as described in “Materials and Methods,” treated with vacuolar ATPase inhibitor Baf A1 and imaged with Zeiss LSM880. Note that before Baf A1 treatment, LysoSensor fluorescence is seen in the EELSs (arrows) due to a low pH environment within their lumen. After treatment with Baf A1 LysoSensor fluorescence is diminished. Scale bars are 10 μm. **(C)** Phase–contrast images of COG4 KO cells before and after drug treatment. EELSs disappear after 24 h of treatment with 200 nm Bafilomycin A1. Scale bars are 50 μm. **(D)** Bar graph indicates the average number of EELSs per cell before and after treatment with Baf A1. **(E)** COG depleted cells are more sensitive to Baf A1 treatment. WT and COG4 KO cells were seeded at 40% confluence and incubated in culture media with or without with Baf A1. 72 h later dishes were rinsed with PBS to remove dead detached cells and remaining cells were counted. Arrows point to EELSs that are between 1 to 10 μm in diameter. Three fields were imaged and error bars indicate SD for *n* = 3. ^∗∗^*p* < 0.01.

During the initial characterization of major morphological phenotypes in HEK293T cells completely depleted for individual COG subunits we observed a significant accumulation of large vacuole-like intracellular structures that were positive for both endosomal and lysosomal markers (Bailey Blackburn et al., 2016). A majority (95%) of Lamp2-positive membranes in wild-type (WT) HEK293T cells are ≤0.91 μm in diameter (Supplementary Figure S1). We called enlarged vacuole-like compartment (≥1 μm in diameter) observed in COG KO cells enlarged endo-lysosomal structures (EELSs). The formation of EELSs was COG-dependent since stable expression of GFP-tagged wild-type copies of the missing COG subunit completely abolished EELS formation (Figure 1A). A significant accumulation of EELSs was also observed in fibroblasts obtained from COG7-CDG patients (Blackburn et al., 2018) as well as in recently created COG4 KOs in human retinal pigment epithelial diploid RPE-1 cells (data not shown). Staining of live HEK293T COG4 KO cells with both LysoSensor (Figure 1B) and LysoTracker (Figure 4B and Supplementary Figure S3), which only fluoresce or are preferentially sequestered in acidic environments, indicated that the lumen of EELSs’ is acidic. Importantly, this pH dependent fluorescence of LysoSensor is lost upon short treatment (4 h) with Baf A1, an inhibitor of vacuolar ATPase (vATPase) (Figure 1B). Interestingly, the EELSs completely disappear after prolonged treatment (24 h) of both COG4 KO and COG7 KO cells with Baf A1 suggesting that the activity of vATPases and the resulting low pH in the lumen of EELSs is necessary for their maintenance (Figure 1C,D).

Bafilomycin A1-induced removal of EELSs from COG4 KO cells renders these cells less viable than WT cells treated with Baf A1 for 72 h (Figure 1E), indicating that the formation of EELSs could be an adaptive feature in cells depleted for COG complex activity.

### Golgi Glycosyltransferases Are Targeted to EELSs

**FIGURE 2:**
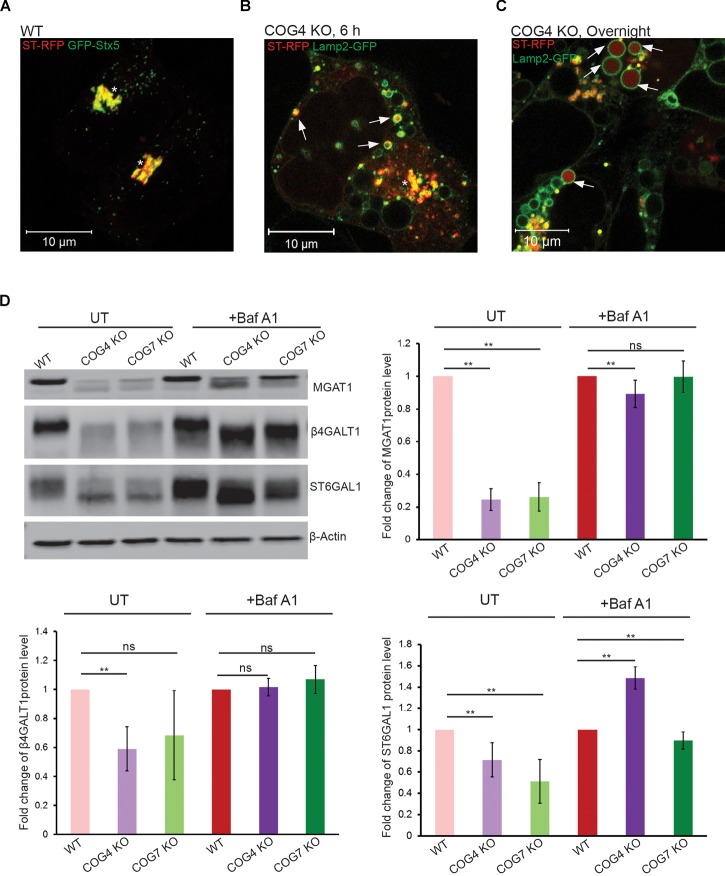
Golgi enzymes partially co-localize with EELSs in COG KO cells and their stability is dependent on the activity of vacuolar ATPase. **(A)** ST-RFP is co-localized with Golgi marker GFP-STX5 in WT HEK 293T cells. **(B)** 6 h after transfection ST-RFP partially co-localizes with Lamp2-GFP positive EELSs in COG4 KO cells (Arrows point to EELSs, star indicates Golgi region). **(C)** After overnight expression in COG4 KO cells RFP fluorescence is seen within Lamp2 positive EELSs (arrows). **(D)** Western blot analysis of three Golgi enzymes in HEK293T cells before and after treatment of cells with 200 nm Baf A1 for 24 h. Bar graphs represent the fold change of Baf A1 treated vs. untreated protein levels. Error bars show SD for *n* = 3 (biological replicates). ^∗∗^*p* < 0.01.

To look at whether EELSs are accessible to Golgi enzymes we transiently expressed RFP-tagged ST6GAL1 (ST-RFP) in COG4 KO cells. This construct is usually strictly Golgi localized in wild-type cells (Figure 2A; Lavieu et al., 2014) but in COG4 KO cells it is rapidly (6 h after expression) relocated to Lamp2-GFP-positive small and enlarged structures (Figure 2B), indicating that Golgi enzymes are mistargeted to endolysosomal compartments in COG KO deficient cells. Even more strikingly, after overnight expression ST-RFP is entirely off the Golgi and the RFP fluorescence is seen within the lumen of Lamp2 positive compartments, including EELSs (Figure 2C), indicating active cleavage/degradation of the transmembrane ST-RFP construct. Supporting this notion, we have previously shown that both medial (MGAT1) and *trans*-Golgi (B4GALT1 and ST6GAL1) enzymes are unstable in COG-deficient cells (Blackburn et al., 2018). To confirm that Golgi enzymes were being degraded in the acidic compartment we used Baf A1 to inhibit vATPase-dependent degradation and re-assessed the stability of these enzymes. After 24 h of Baf A1 treatment we saw improved stability of the underglycosylated Golgi enzymes (Figure 2D). This observation combined with the loss of EELSs upon Baf A1 treatment (Figure 1C) suggested that an acidic lumen within the EELSs may be contributing to pronounced degradation of Golgi enzymes in COG-deficient cells.

### Late Endocytic Markers Are Present on EELSs

**FIGURE 3:**
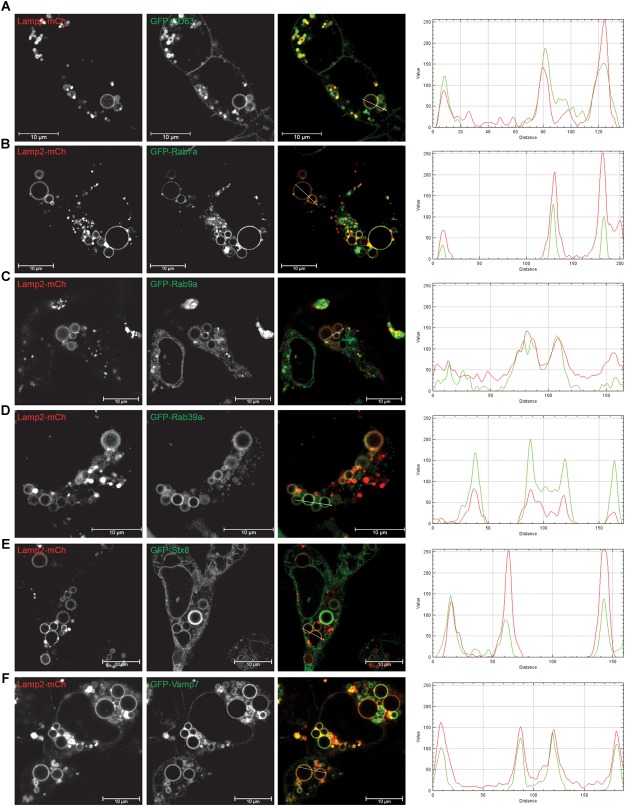
EELSs are positive for late endocytic markers. HEK293T COG4 KO cells were transiently co-transfected with DNA constructs expressing Lamp2-mCherry and **(A)** GFP-CD63, **(B)** GFP-Rab7a, **(C)** GFP-Rab9a, **(D)** GFP-Rab39a, **(E)** GFP-STX8, and **(F)** GFP-Vamp7. Superresolution images were collected using Zeiss LSM880 AiryScan microscope. Graphs of intensity vs. distance represent colocalization between mCh and GFP. Scale bars are 10 μm.

To determine which markers are present on the EELSs we used a superresolution microscopy approach in combination with staining for specific markers of the ER (ER tracer), Golgi (Giantin, GOSR1), PM (VAMP3) and endolysosomal (Rab5, Rab7, Lamp2, and STX8) system (Supplementary Figure S3). EELSs were positively stained with late-endosomal/lysosomal marker Lamp2 (Blackburn et al., 2018) and our initial staining attempts revealed that Lamp2-positive EELSs were also positive for Rab7a and STX8 and negative for Golgi markers Rab6, GOSR1, and Giantin (Supplementary Figure S3). Unfortunately, the EELSs do not preserve well upon PFA fixation, which causes the structures to partially collapse. To work around this, we used transient transfections of fluorescently tagged proteins. To avoid overexpression artifacts, only cells weakly expressing FP-tagged proteins were analyzed (Figure 3 and Supplementary Figure S2). Analysis of HEK293T COG4 KO cells that co-express Lamp2-mCherry and GFP-tagged markers revealed that another endolysosomal marker CD63 (Lamp3) completely co-localized with EELSs (Figure 3A). We also found co-localization of two late-endosomal Rabs; Rab7a, and Rab9a (Figure 3B,C) and Golgi/endosomal Rab39a with Lamp2 on the EELSs’ membrane (Figure 3D). Other tested GFP-tagged Rabs (Rab1, 3, 4, 5, and 6) failed to co-localize with Lamp2-mCherry-positive EELSs (Supplementary Figure S3). Analysis of GFP-tagged SNAREs revealed that only two late endosomal SNAREs, Qa-SNARE STX8 and R-SNARE VAMP7, were present on membranes of EELSs (Figure 3E,F). Other tested GFP-tagged SNAREs (STX16, STX17, Sec22b, GOSR1, Vamp3, Vamp4, Vamp8, SNAP25, and SNAP29) were not localized on the membrane of Lamp2-mCherry-positive EELSs (Supplementary Figure S3).

**FIGURE 4:**
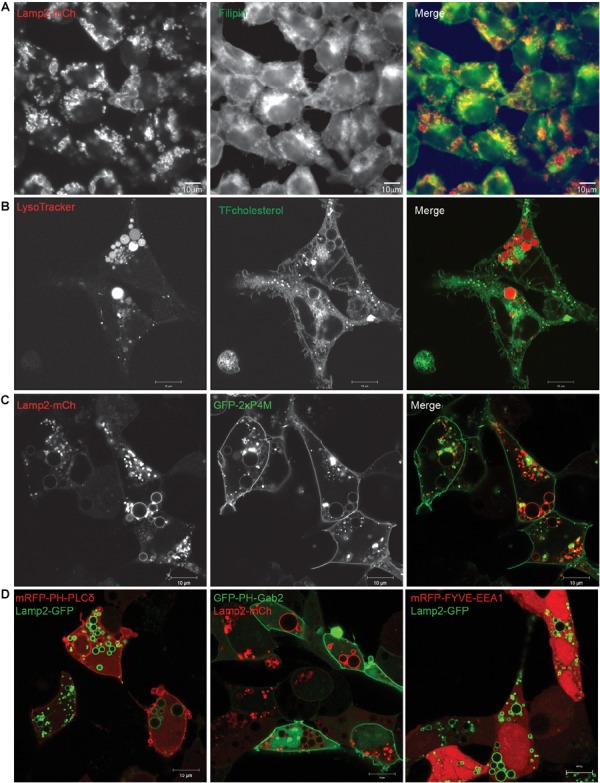
Lipids present in EELSs’ membranes include PI4P and cholesterol. **(A)** Filipin staining in COG4 KO cells shows that EELSs’ membranes have cholesterol. **(B)** Presence of cholesterol was confirmed by TopFluor-cholesterol. EELSs are labeled by LysoTracker **(C)** Phosphatidylinositol PI4P, detected by the biosensor GFP-2×P4M colocalizes with Lamp2-mCh positive EELSs, **(D)** but, EELSs are negative for PI(4,5)P_2_, PI(3,4,5)P_3_, and PI3P detected by biosensors mRFP-PH-PLCδ, GFP-PH-Gab2, and mRFP-FYVE-EEA1, respectively. Scale bars are 10 μm.

Filipin, which forms complexes with cellular cholesterol, labels the Golgi apparatus of fixed cells (Pagano et al., 1989). We found that this fluorescent compound co-localizes with Lamp2-positive EELSs (Figure 4A), indicating that the membrane of EELSs is cholesterol-rich. In support of this finding, incubation of live COG4 KO cells with TopFluor cholesterol revealed a significant distribution of the fluorescent cholesterol analog to the membrane of acidic EELSs (Figure 4B).

To better understand why some molecules from Golgi and endosomal origins are preferentially targeted to the EELSs we decided to look as phosphatidylinositol (PI) species, which are known to be lipid determinants that identify different compartment membranes from one another in the cell. To analyze PI distribution in relation to EELSs, we used lipid biosensors, generously gifted by Dr. Sergio Grinstein (Bohdanowicz et al., 2012; Levin et al., 2016). Interestingly, the EELSs’ membranes were found to be enriched in PI4P (Figure 4C), a lipid normally localized in the *trans*-Golgi and PM (Supplementary Figure S4; Behnia and Munro, 2005; Levin et al., 2017). The EELSs were negative for PI(4,5)P_2_, PI(3,4,5)P_3_, and PI3P (Figure 4D). Taken together, this data suggests that EELSs acquire late endocytic markers, cholesterol and PI4P.

**FIGURE 5:**
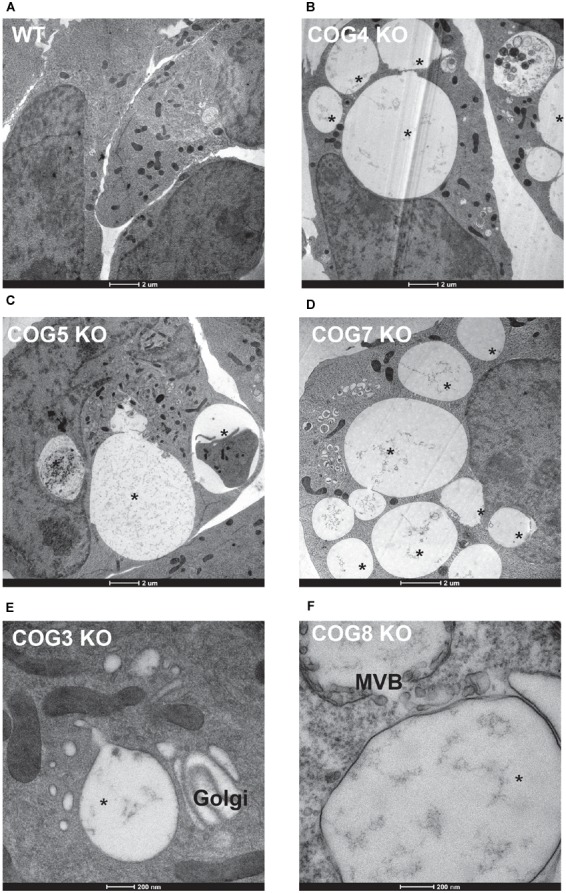
Characterization of the EELSs by Electron microscopy. **(A)** Wild-type and **(B–F)** COG KO HEK293T cells grown on sapphire disks were high pressure frozen, fixed, embedded in Araldite 502/Embed 812 resin and analyzed by transmission electron microscopy. Note that the EELSs (labeled with star) are very prominent vacuole-like compartments in every COG KO cell.

To better understand the EELSs structure and contents, we turned to transmission electron microscopy (Figure 5). Large electron-sparse “empty” vacuole-like membrane structures were visible in every COG-deficient clone analyzed (Figure 5B–F), but were absent in wild-type HEK293T cells (Figure 5A). A small fraction of peripheral vacuoles contain either membrane-like (Figure 5B) or even cell-like (Figure 5C) internalized cargo which may represent phagosomal structures. The EELSs were often located close to the fragmented Golgi (Figure 5D,E) and multi-vesicular bodies (MVBs) (Figure 5F) but were morphologically distinct from both organelles. Additionally, there was no apparent contact between the EELSs and the ER.

**FIGURE 6:**
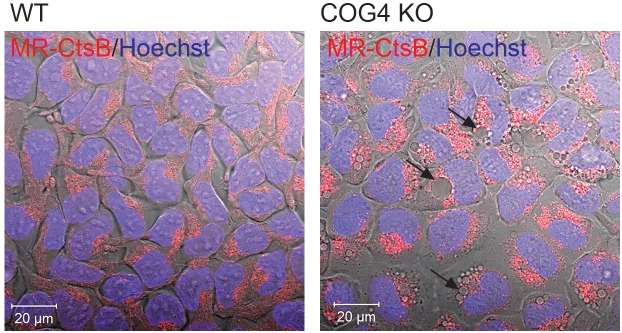
Activity of lysosomal enzyme Cathepsin B is absent in EELSs. HEK293T and COG4 KO cells were incubated with Magic Red substrate for Cathepsin B and Hoechst. Live cell images of DIC, DAPI and mCherry channels were acquired using Zeiss LSM880 microscope. Note the absence of Cathepsin B activity in large EELSs (arrows). Scale bars are 20 μm.

Lamp2 is localized on both late endosomal and lysosomal membranes (Chen et al., 2010). We have previously shown that lysosomal enzyme Cathepsin D is partially missorted and secreted in COG-deficient cells (Blackburn et al., 2018) leading us to wonder if lysosomal enzymes might be mistargeted to these structures. Therefore we looked for the activity of lysosomal the enzyme Cathepsin B within the EELSs using the Magic Red Assay (Figure 6). In this assay, the Magic red substrate fluoresces upon cleavage by Cathepsin B making it a read out for localization of active Cathepsin. Overall intracellular activity of Cathepsin B was similar in HEK293T WT and COG4KO cells indicating that function of lysosomes was not dramatically disturbed in COG-depleted cells. Interestingly, enzyme activity was absent in EELSs indicated by the absence of red fluorescence which is robust in normal sized lysosomes of both WT and COG4 KO cells suggesting that the EELSs, though acidic and positive for lysosomal markers such as Lamp2 and Rab7a are distinct from mature lysosomes (Figure 6).

**FIGURE 7:**
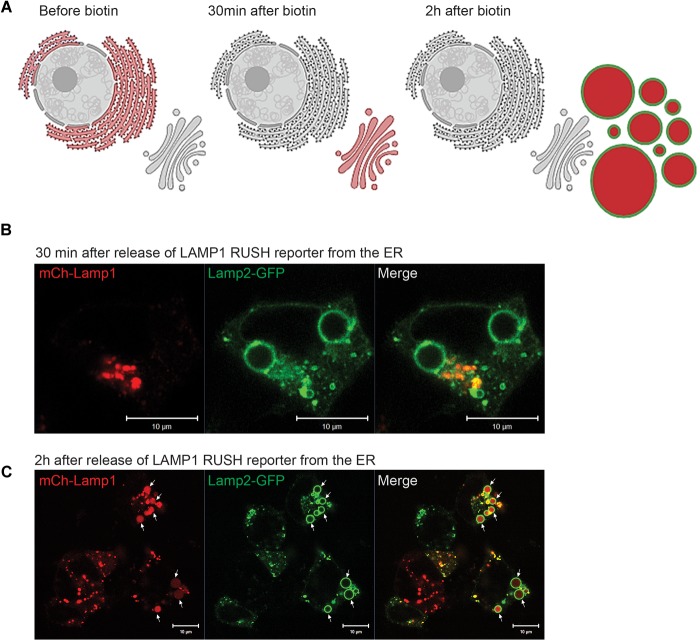
Biosynthetic cargo is delivered to EELSs with fast kinetic. **(A)** Schematic representation of the RUSH system experiment using reporter SBP-mCherry-Lamp1. **(B)** 30 min after release from ER mCherry-Lamp1 is localized in perinuclear Golgi region distinct from Lamp2-GFP-positive EELS. **(C)** After 120 min chase majority of mCherry-Lamp1 is seen within Lamp2-GFP EELSs. Superresolution images were collected using Zeiss LSM880 AiryScan microscope. Scale bars are 10 μm. Arrows indicate Lamp1 and Lamp2 positive EELSs.

We next asked how long it would take for newly synthesized biosynthetic cargo to get to EELSs. Since our initial experiments utilizing VSVG-ts45 system (Hirschberg et al., 1998) did not reveal any significant transport of plasma membrane-localized transmembrane cargo to or through the EELSs (data not shown), we used an alternative assay with endolysosome-specific cargo, mCherry-Lamp1 using the RUSH system. The RUSH system was designed for synchronous biotin-dependent release of the reporter from the ER allowing visualization of the fate of the reporter as it passes through the secretory pathway (Boncompain et al., 2012). Figure 7A shows a schematic depiction of the RUSH assay. We chose to use Lamp2-GFP in addition with the Lamp1 RUSH construct as endogenous Lamp1 colocalizes with Lamp2 in a steady-state (Raposo et al., 1997). HEK293T COG4 KO cells were co-transfected with the Lamp1 RUSH reporter and Lamp2-GFP overnight in biotin-free media to allow for the accumulation of mCherry-Lamp1 in the ER and delivery of Lamp2-GFP to the EELSs. Biotin and cycloheximide were added 16 h after transfection to release mCherry-Lamp1 and to block any additional protein synthesis. 30 min after biotin addition mCherry fluorescence of the Lamp1 RUSH reporter was mostly perinuclear and distinct from Lamp2-GFP, indicating that RUSH reporter had reached the Golgi (Figure 7B). About 90 min later, mCherry signal starts to appear within Lamp2-GFP positive EELSs. At 2 h clear localization is seen within EELSs (Figure 7C) confirming that selective biosynthetic cargo is delivered to this compartment. Interestingly, majority of mCherry signal was localized not on the EELSs’ membrane labeled with Lamp2-GFP, but instead in the EELSs’ lumen indicating that either whole mCherry-Lamp1 molecule was internalized within EELSs or that mCherry was cleaved from the mCherry-Lamp1 construct. It is important to note that GFP in the Lamp2-GFP construct is localized on the cytoplasmic side of the membrane, while mCherry in mCherry-Lamp1 construct is predicted to be on the luminal side, thus potentially exposing it to cleavage in the acidic environment of the EELSs.

### Endocytic Cargo Accumulates Within EELSs

**FIGURE 8:**
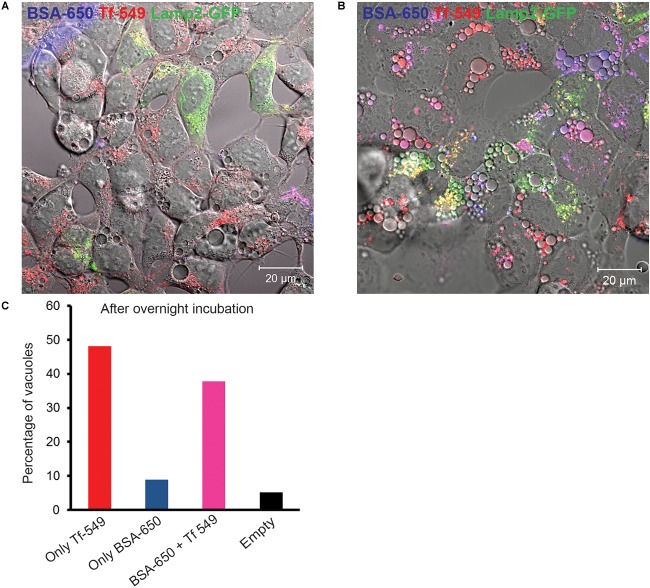
Endocytic cargo is delivered to EELSs with slow kinetic. HEK293T COG4 KO cells were incubated with both bulk phase endocytic/pinocytic (BSA-650) and receptor mediated (Transferrin, Tf-549) markers for **(A)** 1 h and for **(B)** 24 h. Note that after 1 h of feeding, Tf and BSA enter endosomal compartments but are completely absent in the EELSs. Only after 24 h incubation, fluorescently labeled endocytic cargo BSA-650 and Tf-549 accumulate within EELSs. **(C)** Bar graph represents the number of EELSs in the field shown in **(B)** that have accumulated either Tf-650 or BSA-650 or both Tf and BSA. Images were collected using Zeiss LSM880 microscope. Scale bars are 10 μm.

Having established that newly synthetized Lamp1 is rapidly delivered to EELSs we turned to analysis of the endocytic cargo. HEK293T COG4 KO cells were fed with fluorescently labeled BSA-650 and Tf-549 to see whether receptor mediated (Tf), (Harding et al., 1983) or bulk (BSA) (Harding et al., 1985) endocytic cargo gets delivered to EELSs. Prior to feeding with endocytic tracers, these cells were transfected with Lamp2-GFP to label the EELSs. After 1 h incubation with BSA-650 and Tf-549, both markers appear in intracellular puncta that are likely to represent normal endosomes/lysosomes but do not yet appear in or on EELSs (Figure 8A). However, after 24 h accumulation of both fluid-phase and receptor-mediated tracers can be seen within EELSs (Figure 8B), indicating that EELSs are accessible to endocytic cargo, however, with a slow rate of delivery. Interestingly, at this time point the distribution of endocytic markers in EELSs was heterogeneous (Figure 8C). Majority (more than 90%) of EELSs were positive for Tf, while only half of them contained both BSA and Tf and a very small percentage was either empty or only BSA-positive. Though Tf and BSA are internalized via different mechanisms since COG4 KO cells were simultaneously fed with BSA and Tf, some fraction of Tf would also enter via bulk phase endocytosis/pinocytosis making the heterogeneous distribution into EELSs rather surprising and raising the question of whether there is any selectivity that directs the delivery of endocytic cargo to the EELSs. In conclusion, EELSs are accessible to both fluid-phase and receptor-mediated cargo, but the delivery of both types of cargo is delayed in comparison to the rate of delivery of biosynthetic cargo (ST6Gal1 and Lamp1 RUSH reporter).

**FIGURE 9:**
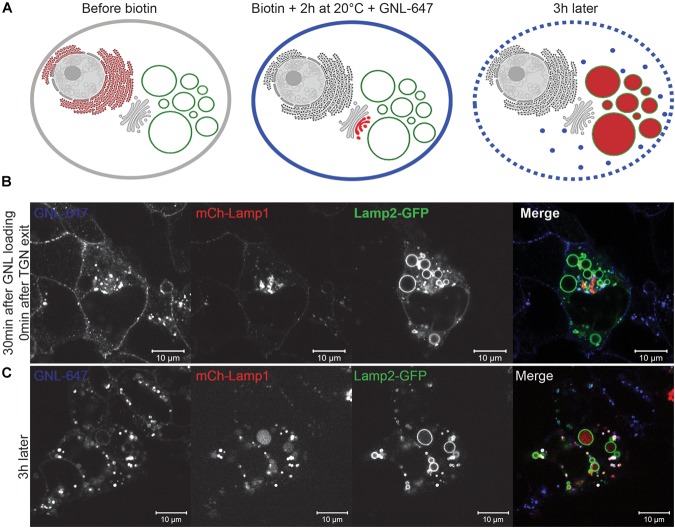
EELSs’ cargo is preferentially delivered from the Golgi. 20°C block was utilized to accumulate the RUSH reporter mCherry-Lamp1 in TGN of HEK293T COG4 KO cells. After that, plasma membrane glycoproteins were labeled with GNL-647 and delivery of both endocytic and biosynthetic cargo to the EELSs was analyzed using AiryScan microscopy. **(A)** Schematic representation of the RUSH assay with a 20°C TGN exit block. **(B)** Fluorescent images of GNL-647, Cherry-Lamp1 and Lamp2-GFP (EELS marker) immediately after shifting the cells to 37°C and **(C)** after 3 h of shifting the cells to 37°C. Scale bars are 10 μm.

### The EELSs Preferentially Originate From the Golgi

In order to compare the kinetics of the delivery of biosynthetic and endocytic cargo to EELS directly we modified the RUSH system by introducing an additional TGN exit block. COG4 KO cells were co-transfected with the mCherry-Lamp1 RUSH reporter and Lamp2-GFP. The next day, immediately after adding biotin and cycloheximide, the cells were incubated at 20°C for 2 h to induce a TGN exit block essentially trapping the RUSH construct in the TGN (Griffiths et al., 1989). Additionally, all plasma-membrane glycoproteins were decorated with fluorescently labeled GNL-647, a lectin which binds to immature surface glycans in in COG KO cells (Bailey Blackburn et al., 2016; Blackburn and Lupashin, 2016). This set up essentially enabled simultaneous tracking of biosynthetic and endocytic cargo as well as the comparison of the kinetics of delivery. Figure 9A schematically depicts the experimental set up. At the beginning of the chase, GNL-647 signal is localized on the plasma membrane as well as in endosomes and mCherry signal of Lamp1 RUSH reporter is perinuclear in the TGN (Figure 9B). The TGN localization of the RUSH reporter was confirmed by immunofluorescence with a TGN marker, TGN46 (data not shown). 3 h after transferring the cells to 37°C, all mCherry signal is seen within the lumen of Lamp2 positive EELSs but, these are mostly negative for GNL-647 (Figure 9C) confirming that anterograde delivery of Golgi cargo is faster than retrograde delivery of plasma membrane cargo to the EELSs and suggests that the TGN is the dominant supplier of membrane and cargo to the EELSs.

**FIGURE 10:**
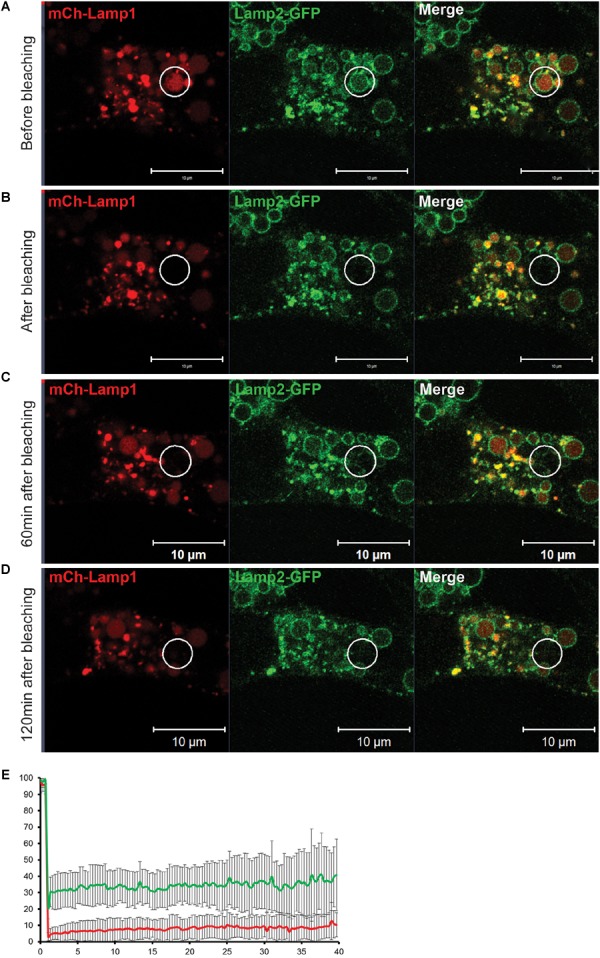
Fluorescent Recovery After Photobleaching (FRAP) experiment reveals that an EELS is a stable organelle that exchanges its membrane and soluble content at a very slow rate. **(A)** Both soluble (mCherry-Lamp1) and membrane (Lamp2-GFP) cargo in EELSs HEK293T COG4 KO cells was simultaneously bleached using 488 nm and 546 nm lasers and recovery of fluorescent signal was analyzed at **(B)** 0 min, **(C)** 60 min, and **(D)** 120 min after the bleach. Superresolution images were collected using Zeiss LSM880 AiryScan microscope. Scale bars are 10 μm, white circles indicate the bleached region **(E)** The graph represents percentage of mCh (red) and GFP (green) signals over the course of 40 min. Error bars indicate SD of five fields that were imaged, 1–2 EELSs were chosen in every field.

**FIGURE 11:**
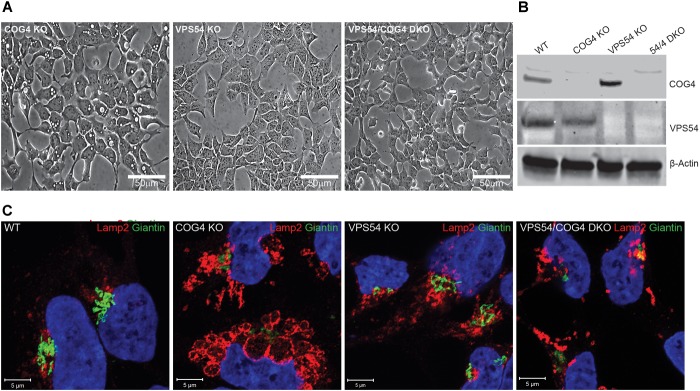
Activity of the GARP complex is necessary for the formation and maintenance of EELSs. **(A)** HEK293T, VPS54 KO, and VPS54/COG4 DKO were created and analyzed for the presence of EELSs using phase-contrast microscopy. Scale bars are 50 μm. **(B)** Absence of knocked-out gene product was analyzed by Western Blot. **(C)** Airyscan images of WT, COG4 KO, VPS54 KO, and VPS54/COG4 DKO stained with the Golgi marker Giantin and late endo/lysosomal marker Lamp2. Lamp2 stained EELSs are only present in COG4 KO cells. Scale bars are 5 μm.

### An EELS Is a Stable Hybrid Compartment

The appearance of EELSs is a very prominent feature of COG-depleted cells; at the same time, they disappear after Baf A1 treatment and during chemical fixation, suggesting their fragile and/or transient nature. Live cell analysis indicated that EELSs exhibit slow movement within cells and are not as dynamic as endosomes/lysosomes, but both membrane and soluble cargo may be exchanged between EELSs and other organelles of secretory and endocytic pathways. To test this we employed a fluorescent recovery after photobleaching (FRAP) approach on cells transfected with Lamp2-GFP (membrane cargo) and mCherry-Lamp1 (soluble cargo) to analyze the rate of the material exchange between EELSs and other organelles. Both GFP and mCherry fluorescence within one EELS in HEK293T COG4 cells co-expressing Lamp2-GFP (membrane cargo) and mCherry-Lamp1 (soluble cargo) was bleached and the time course of fluorescence recovery was analyzed (Figure 10). Strikingly, no significant fluorescence recovery of both membrane and soluble markers was detected even after 2 h indicating that the membranes of EELSs do not physically connected to other membranes of secretory and endocytic pathways and that fusion with upcoming membrane transport carriers happens at a slow rate. A similar result was observed while performing FRAP analysis only with luminal mCherry (Supplementary Figure S5). It is important to note, that in this experiment Lamp2-GFP-positive EELSs remained intact ruling out the possibility of the EELS collapsing upon photobleaching. We concluded that the EELS is a stable membrane compartment with restricted communication with other secretory and endocytic compartments.

### GARP Activity Is Necessary for EELSs Formation

To test if the deficiency of another Golgi vesicle tethering complex, GARP, will result in accumulation of EELSs we knocked out VPS54 (Figure 11) and VPS53 (data not shown) in HEK293T cells. VPS54 is a unique subunit of the GARP complex, an MTC that operates between late endosomes and TGN (Bonifacino and Hierro, 2011) while VPS53 belongs to both GARP and EARP (Endosome-Associated Recycling Protein) complexes (Schindler et al., 2015). In both KO cells lines (Figure 11A and data not shown) no accumulation of enlarged vacuole-like structures was detected, indicating that EELSs are COG KO specific and are formed due to trafficking defects caused by loss of COG’s function. Interestingly, knock out of VPS54 in COG KO cells (VPS54/COG4 DKO) resulted in a complete disappearance of the EELSs and the cells appeared like HEK293T WT or COG rescued cells in shown in Figure 1, 11C). Similar results were obtained with VPS53/COG4 DKO cells (Supplementary Figure S6). This led us to conclude that the EELSs’ formation requires the activity of the GARP complex in COG deficient cells.

## Discussion

In this study, we continue our investigation of a specific phenotypic defect that arises upon complete depletion of individual COG subunits. Using microscopy and biochemistry approaches, EELSs were characterized to understand how they are formed in COG KO HEK293T cells. This phenotype is not unique to HEK293T cells and is present in COG4 KO RPE1 (data not shown) cells and in a subset of fibroblasts obtained from COG7-CDG patients (Blackburn et al., 2018).

**FIGURE 12:**
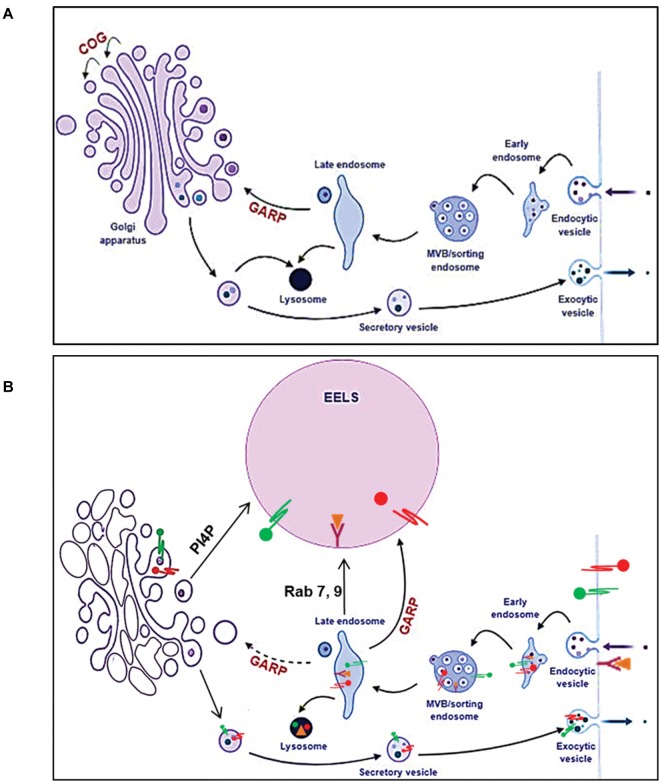
Model of altered intracellular membrane trafficking in COG depleted cells. In normal cells **(A)**, the balance between the anterograde and retrograde trafficking ensures maintenance of compartment identity and proper trafficking of secretory cargo. Once secretory cargo reaches the end of the Golgi, it undergoes sorting and packaging into vesicles targeted to appropriate compartments. Endosomes containing endocytic cargo go thorough different stages of endosomal maturation and become late endosomes. Cargo form late endosomes is delivered to the Golgi. The GARP complex tethers late endosomes to the TGN. Golgi resident proteins and enzymes such as glycosyltransferases that get packaged along with secretory cargo are cycled back to the Golgi from late endosomes. Additionally, late endosomes also deliver lysosomal proteases and cargo destined for degradation to lysosomes. **(B)** When any individual COG subunit is knocked out, the COG complex is non-functional consequently affecting retrograde trafficking at the Golgi. However, the active GAPR complex, tethers late endosomes to the TGN feeding it with cargo and membrane. This results in a bottle neck effect at the TGN and the consequent enlargement of this compartment manifests as EELSs. Hence, when the GAPR complex is no longer active in COG4 KO cells, the EELSs are no longer formed. This figure was made using BioRender.

Our studies revealed that formation of EELSs is COG specific and this phenotype is rescued upon stable re-expression of the WT copy of the deleted COG subunit. Importantly, formation and/or stability of EELSs were also severely diminished in cells treated with vacuolar-ATPase inhibitor Baf A1 and upon inhibition of another MTCs GARP. Based on these observations we hypothesized that formation of EELSs depends on the activity of vATPase and that GARP is playing an essential role in delivery of membranes to EELSs because knocking out VPS54 results in the loss of EELSs (Figure 11, 12). In agreement to this prediction we found that the lumen of EELSs is acidic due to the activity of vATPase. vATPases pump protons into the lumen of organelles thus maintaining an appropriate pH in their lumen (Oot et al., 2017). The decreasing pH gradient from *cis* to *trans*-Golgi is also maintained by the activity of vATPases (Marshansky and Futai, 2008). This pH gradient is crucial for functions within the Golgi like glycosylation and cargo sorting to be properly executed.

Impairment in one of the subunits of v-ATPase, the a2 subunit, causes a type II-CDG, the same subtype of CDG that COG depletion causes (Kornak et al., 2008; Bahena-Bahena et al., 2014). This subunit anchors vATPases into membranes and also provides a channel for protons to move from the cytosol to the lumen. One possibility is that the COG complex machinery is responsible for proper targeting/retention of vATPase in the Golgi sub-compartments and COG malfunction alters vATPase’s intracellular distribution creating a hybrid post-Golgi compartment with very low pH. Our preliminary mass-spectrometry analysis of COG complex membrane partners indicates that several subunits of vATPase may indeed directly or indirectly interact with COG4 and COG8 (JBB, VL, personal communication).

We have also observed that EELSs are lost in cells under stress induced by various triggers such as overnight treatment with cycloheximide or starvation (ZD, VL, personal communication). One possibility is that the loss of EELSs could be due to the disassembly of vATPase under stress to conserve cytosolic ATP (Kane, 2000). However, this is only conjecture and a detailed analysis of vATPases in COG KOs will shed more light. At this point it is clear that low pH with the lumen of EELSs is crucial for their maintenance. The relatively large size (up to 10 μm in diameter) and “empty” appearance of the EELSs (Figure 5) indicate that in addition to vATPase other ion and water transporters may also be mistargeted to EELSs in COG deficient cells. It will be important to test if aquaporins that reside constitutively at the plasma membrane in most cell types are redistributed to EELSs. Several studies have demonstrated that the aquaporins are present in intracellular vesicles in liver and kidney, implying that aquaporins in post-Golgi compartments could be involved in their volume regulation (Sugiya et al., 2008). If aquaporins are present on EELSs, it would also explain why EELSs are sensitive to disruption of H^+^ homeostasis which is lost upon BafA treatment. In this scenario, aquaporins may constantly pump in water to maintain osmotic balance between EELSs’ acidic lumen and the cytosol. Baf A1 induced vATPase inactivation makes the EELSs hypotonic with respect to the cytosol and so the EELSs collapse because of outward movement of water.

What is the origin of EELSs? Appearance of the EELSs in COG deficient cells is a very unusual and an unexpected cellular phenotype. However, formation of enlarged endocytic compartments has been observed previously as a result of malfunctioning of endocytic sorting/trafficking machinery (Choudhury et al., 2005; Kamalesh et al., 2017; Kaur et al., 2018). An example of this is the appearance of enlarged early endosomes in cells expressing GTP-restricted Rab5 (Stenmark et al., 1995). To our knowledge, our lab is the first to implicate altered Golgi trafficking machinery in the formation of the enlarged post-Golgi acidic compartments. We believe that the EELSs actually originate from the *trans*-Golgi in COG-deficient cells as a result of protein and lipid mis-targeting. Cargo delivery studies (Figure 7, 8) provide the evidence that EELSs are dominantly fed by anterograde trafficking and the delivery of endocytic cargo is much slower in comparison. We have found that EELSs are enriched in cholesterol, similar to the TGN and plasma membrane and typical TGN lipid, PI4P (Figure 4). Interestingly, the localization of yeast vATPases was shown to be regulated by its interaction with a PI4P (Banerjee and Kane, 2017). The EELSs are enriched with PI4P and this interaction may be the reason EELSs acquire vATPases. Besides PI4P no other Golgi marker co-localized with EELSs by IF, suggesting that Golgi proteins are either rapidly degraded as in the case of ST6GAL1 (Figure 2A), within the acidic lumen of EELSs or is efficiently sorted out of EELSs. Efficient sorting out of resident proteins should include robust communication between the EELSs and other cellular compartments, but our FRAP experiments indicate very limited exchange for both soluble and transmembrane cargo. Interestingly, transmembrane protein mCherry-Lamp1 was efficiently delivered to EELSs, but mCherry fluorescence was detected mostly in the EELS’s lumen, indicating degradation or specific cleavage of this hybrid molecule. It will be interesting to investigate which enzyme actually cleaves the mCherry-Lamp1 hybrid since tested lysosomal proteases like Cathepsin B were mostly absent from EELSs (Figure 6). EELSs are positive for a subset of late endosomal markers (Rabs7, 9, 39, Lamp1, 2, CD63, STX8, and Vamp7) and we hypothesized that these proteins are delivered to nascent EELSs in a GARP-dependent mechanism (Figure 12). In this scenario defects in GARP-dependent delivery would abolish “feeding” and enlargement of nascent EELSs, essentially rescuing EELSs’ formation. Based on distribution of endocytic cargo and EM analysis we also propose that the EELSs are heterogeneous and include large phagocytic vacuoles directly originating from plasma membrane and containing large extracellular cargo and even neighboring cells (Figure 5C). In the future it will be important to figure out what triggers the increase of phagocytic activity in COG deficient cells.

What is the function of EELSs in COG deficient cells? The localization of a COG sensitive glycosyltransferase ST6Gal1 to enlarged vacuoles (Figure 2A) and the rescue of stability of several Golgi enzymes upon treatment with BafA1 (Figure 2B,C) suggests that these Golgi resident proteins could be degraded in the EELSs. We propose that the formation of EELSs is an adaptive function in COG deficient cells. In favor of this hypothesis, we have observed that Baf A1 induced abolition of the EELSs makes COG4 KO cells less viable that WT treated cells (Figure 1E). The EELSs may serve to isolate and degrade both underglycosylated and missorted proteins and lipids to avert their negative impact on cell physiology.

## Data Availability

All datasets generated for this study are included in the manuscript and/or the Supplementary Files.

## Author Contributions

ZD, JB, and VL designed and conducted the experiments and wrote the manuscript. TK and IP designed and conducted the experiments and edited the manuscript.

## Conflict of Interest Statement

The authors declare that the research was conducted in the absence of any commercial or financial relationships that could be construed as a potential conflict of interest.

## Supplementary Material

FIGURE S1 Scatter plot indicating size distribution of Lamp2 positive compartments in WT and COG4 KO cells. The diameter of Lamp2 positive structures was analyzed in 31 WT cells and 43 COG4 KO cells, transiently transfected with fluorescent protein tagged Lamp2. Of the 724 Lamp2 positive structures in WT cells, 95% of the vacuoles had a diameter ≤0.91 μm while in the case of COG4 KO cells, of the 885 vacuoles counted, 58% of the vacuoles had a diameter ≥0.91 μm. Out of the 58% of EELSs with a diameter ≥0.91 μm, 88% of them had a diameter ≥1 μm which we defined as EELSs.

FIGURE S2 Localization of GFP-CD63 **(A)**, GFP-Rab7a **(B)**, GFP-Rab9a **(C)**, GFP-Rab39a **(D)**, GFP-Stx8 **(E)**, and GFP-Vamp7 **(F)** in WT and COG4 KO cells. Graphs generated using the RGB Profiler plug-in in ImageJ represent the quantification of mCh and GFP signal intensities along the white line (left to right) drawn. Overlapping peaks indicate colocalization of the two markers. Scale bars are 10 μm.

FIGURE S3 ER, Golgi and early endosomal markers do not colocalize with the EELSs. Live cell imaging of COG4 KO cells indicate that EELSs, labeled with LysoTracker Red DND-99 do not colocalize with ER tracker. Immunostaining with Lamp2 antibody or transient transfection with Lamp2-mCh in COG4 KO cells to label EELSs show that EELSs are negative for Golgi (Giantin, TGN38) and Rabs (Rab1, 3, 4, 5, and 6). EELSs are also negative for the SNARES- Stx16, Stx17, GOSR1, Sec22b, SNAP23, SNAP29, Vamp3, Vamp4, and Vamp8. Scale bars are 10 μm.

FIGURE S4 PI4P localization in HEK293T WT cells. GFP fluorescence of the PI4P biosensor, GFP-2×P4M is seen on the PM and has perinuclear/Golgi localization. Scale bars are 10 μm.

FIGURE S5 EELSs are stable after photobleaching. Bleaching of luminal mCh does not result in collapse of the EELS. The cells were co-transfected with the mCh-Lamp1 RUSH reporter construct and Lamp2-GFP. Bleaching only luminal mCh does not affect the EELS’s stability and the EELS can be seen with only GFP on its membrane even 50 min after bleaching. In this time period, there is no recovery of mCh fluorescence. Scale bars are 2 μm.

FIGURE S6 VPS53 KO recues the formation of EELSs in COG4 KO cells. Knocking out VPS53 in COG4 KO cells using a CRISPR-Cas9 mediated approach results in DKO cells with no EELSs. Scale bars are 50 μm.

## Abbreviations

Baf A1: bafilomycin A1
Tf: transferrin
CDG: congenital disorder of glycosylation
COG: conserved oligomeric golgi
EELSs: enlarged endolysosomal structures
ER: endoplasmic reticulum
GARP: golgi-associated retrograde protein
MTC: multi-subunit tethering complex
PM: plasma membrane
RUSH: retention using selective hooks
SNARE: SNAP (soluble NSF attachment protein) receptor
TGN: *trans*-golgi network
VPS: vacuolar protein subunit.
