# Supplementary figures and images for: Defects in COG-Mediated Golgi Trafficking Alter Endo-Lysosomal System in Human Cells
Source: Front Cell Dev Biol. 2019 Jul 3;7:118. doi: 10.3389/fcell.2019.00118 (PMC6616090; doi:10.3389/fcell.2019.00118)

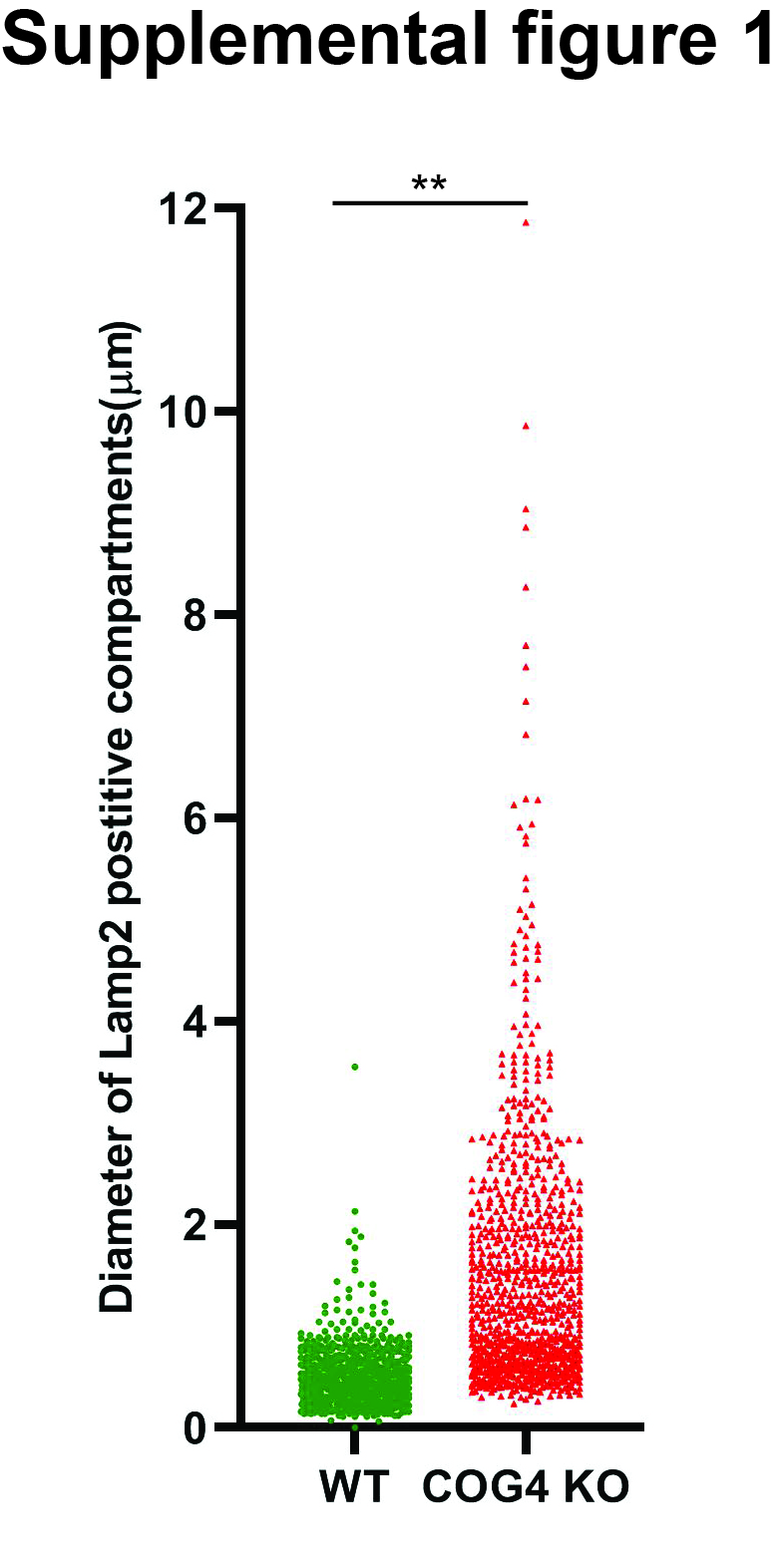

Supplement: FIGURE S1 — Scatter plot indicating size distribution of Lamp2 positive compartments in WT and COG4 KO cells. The diameter of Lamp2 positive structures was analyzed in 31 WT cells and 43 COG4 KO cells, transiently transfected with fluorescent protein tagged Lamp2. Of the 724 Lamp2 positive structures in WT cells, 95% of the vacuoles had a diameter ≤0.91 μm while in the case of COG4 KO cells, of the 885 vacuoles counted, 58% of the vacuoles had a diameter ≥0.91 μm. Out of the 58% of EELSs with a diameter ≥0.91 μm, 88% of them had a diameter ≥1 μm which we defined as EELSs. [file Image_1.JPEG]

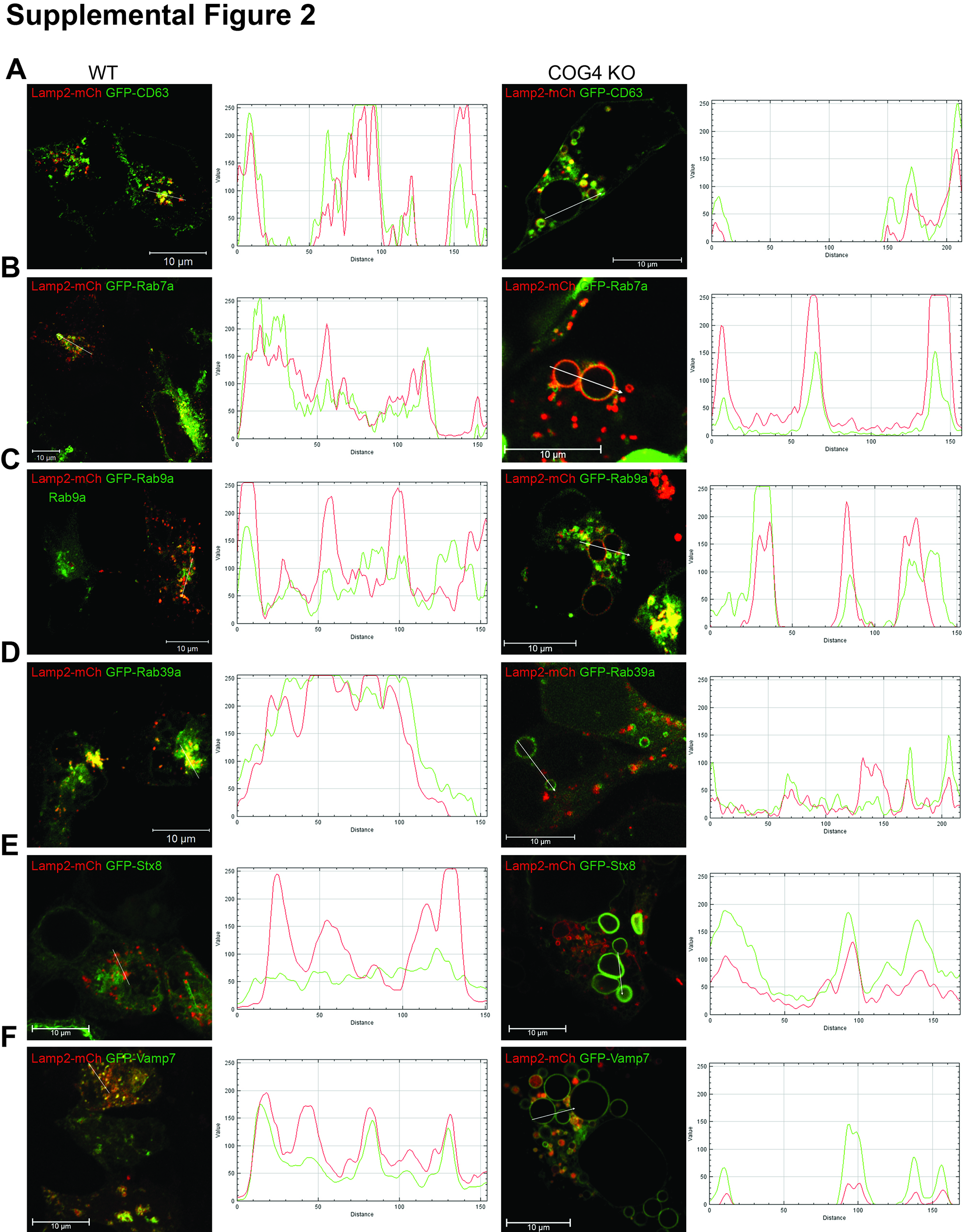

Supplement: FIGURE S2 — Localization of GFP-CD63 (A), GFP-Rab7a (B), GFP-Rab9a (C), GFP-Rab39a (D), GFP-Stx8 (E), and GFP-Vamp7 (F) in WT and COG4 KO cells. Graphs generated using the RGB Profiler plug-in in ImageJ represent the quantification of mCh and GFP signal intensities along the white line (left to right) drawn. Overlapping peaks indicate colocalization of the two markers. Scale bars are 10 μm. [file Image_2.JPEG]

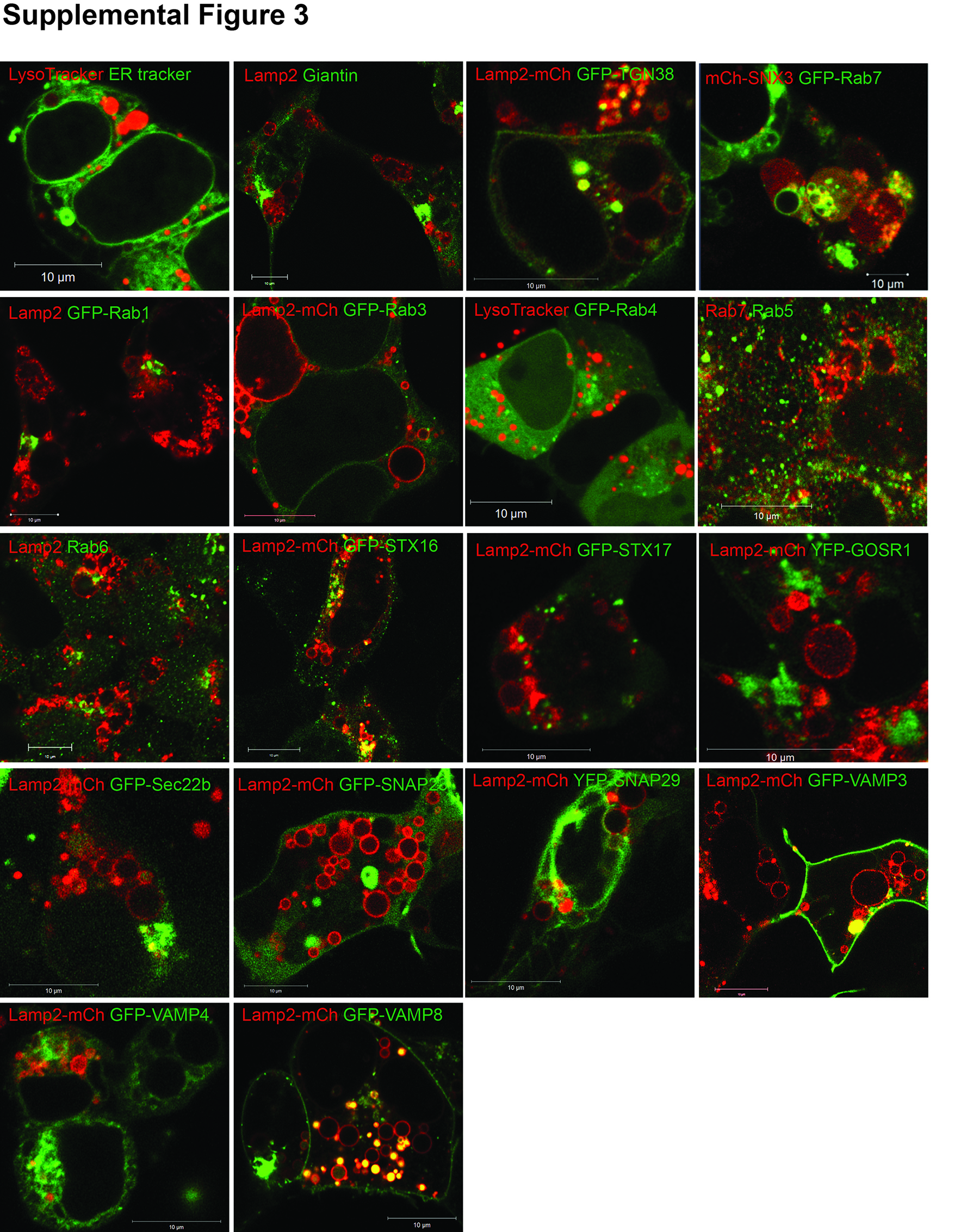

Supplement: FIGURE S3 — ER, Golgi and early endosomal markers do not colocalize with the EELSs. Live cell imaging of COG4 KO cells indicate that EELSs, labeled with LysoTracker Red DND-99 do not colocalize with ER tracker. Immunostaining with Lamp2 antibody or transient transfection with Lamp2-mCh in COG4 KO cells to label EELSs show that EELSs are negative for Golgi (Giantin, TGN38) and Rabs (Rab1, 3, 4, 5, and 6). EELSs are also negative for the SNARES- Stx16, Stx17, GOSR1, Sec22b, SNAP23, SNAP29, Vamp3, Vamp4, and Vamp8. Scale bars are 10 μm. [file Image_3.JPEG]

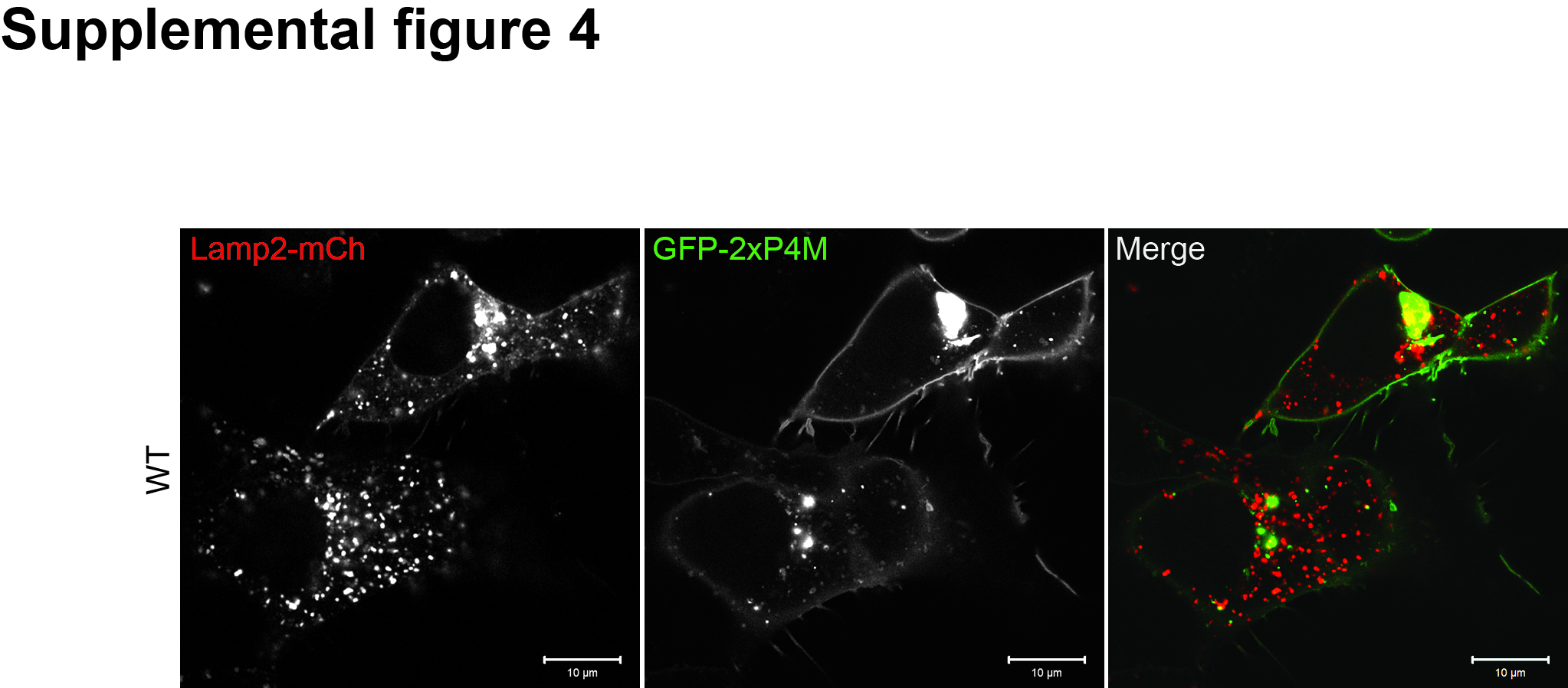

Supplement: FIGURE S4 — PI4P localization in HEK293T WT cells. GFP fluorescence of the PI4P biosensor, GFP-2×P4M is seen on the PM and has perinuclear/Golgi localization. Scale bars are 10 μm. [file Image_4.JPEG]

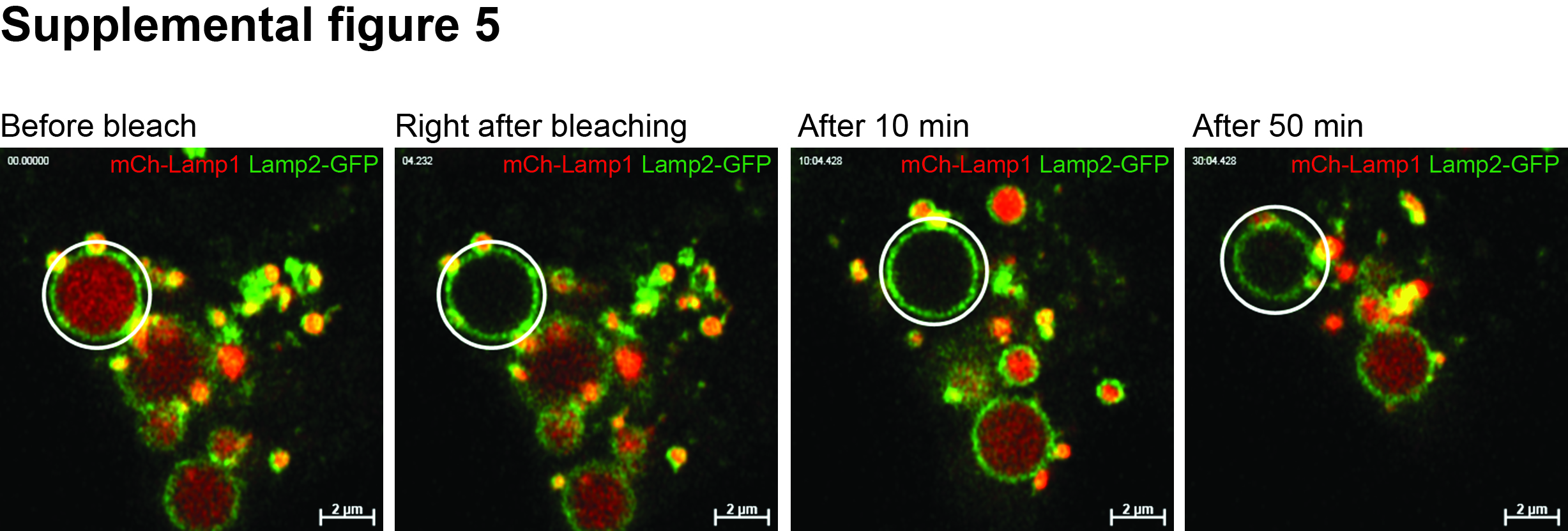

Supplement: FIGURE S5 — EELSs are stable after photobleaching. Bleaching of luminal mCh does not result in collapse of the EELS. The cells were co-transfected with the mCh-Lamp1 RUSH reporter construct and Lamp2-GFP. Bleaching only luminal mCh does not affect the EELS’s stability and the EELS can be seen with only GFP on its membrane even 50 min after bleaching. In this time period, there is no recovery of mCh fluorescence. Scale bars are 2 μm. [file Image_5.JPEG]

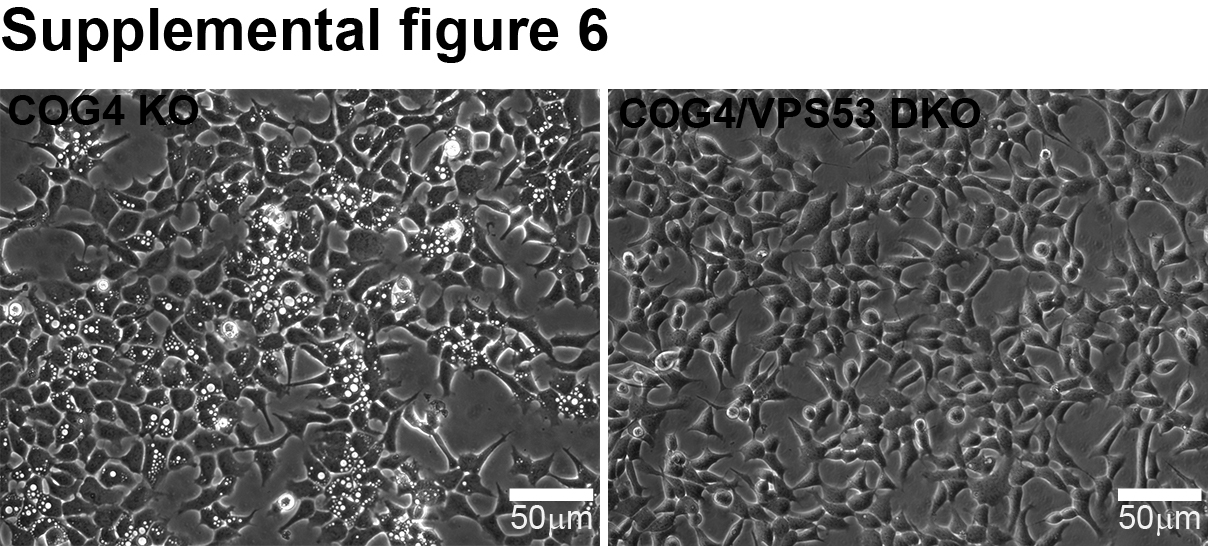

Supplement: FIGURE S6 — VPS53 KO recues the formation of EELSs in COG4 KO cells. Knocking out VPS53 in COG4 KO cells using a CRISPR-Cas9 mediated approach results in DKO cells with no EELSs. Scale bars are 50 μm. [file Image_6.JPEG]
